# Observation of Van Hove Singularities and Temperature Dependence of Electrical Characteristics in Suspended Carbon Nanotube Schottky Barrier Transistors

**DOI:** 10.1007/s40820-017-0171-3

**Published:** 2017-12-13

**Authors:** Jian Zhang, Siyu Liu, Jean Pierre Nshimiyimana, Ya Deng, Xiao Hu, Xiannian Chi, Pei Wu, Jia Liu, Weiguo Chu, Lianfeng Sun

**Affiliations:** 1 CAS Key Laboratory of Nanosystem and Hierarchical Fabrication, CAS Center for Excellence in Nanoscience, National Center for Nanoscience and Technology, Beijing, 100190 People’s Republic of China; 2 University of Chinese Academy of Sciences, Beijing, 100049 People’s Republic of China

**Keywords:** Carbon nanotube, Van Hove singularities, Schottky barrier transistors

## Abstract

A Van Hove singularity (VHS) is a singularity in the phonon or electronic density of states of a crystalline solid. When the Fermi energy is close to the VHS, instabilities will occur, which can give rise to new phases of matter with desirable properties. However, the position of the VHS in the band structure cannot be changed in most materials. In this work, we demonstrate that the carrier densities required to approach the VHS are reached by gating in a suspended carbon nanotube Schottky barrier transistor. Critical saddle points were observed in regions of both positive and negative gate voltage, and the conductance flattened out when the gate voltage exceeded the critical value. These novel physical phenomena were evident when the temperature is below 100 K. Further, the temperature dependence of the electrical characteristics was also investigated in this type of Schottky barrier transistor.

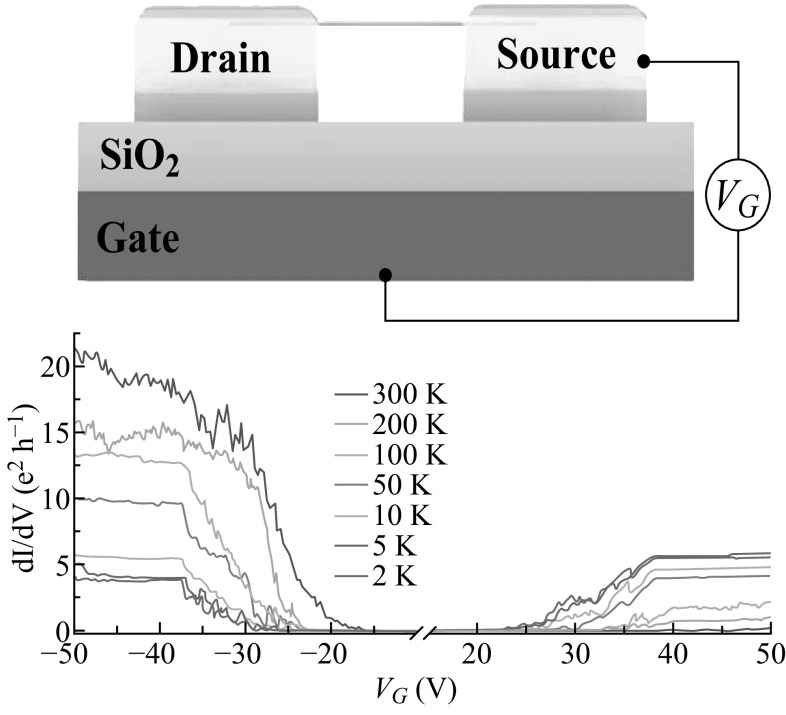

## Highlights

- A clear signature of VHSs in the conductance versus gate voltage was observed in Schottky barrier transistors with an individual suspended single-walled carbon nanotube (SWNT).
- Critical saddle points appear in regions of both positive and negative gate voltage, and the conductance flattens out when the gate voltage exceeds the critical value.

## Introduction

Single-walled carbon nanotubes (SWNTs) are quasi-one-dimensional (1D) materials with semiconducting or metallic properties that make them attractive for both fundamental science and technology [1–3]. SWNTs are also promising materials for future nanoscale device applications such as field-effect transistors (FETs) [4–7]. In conventional SWNT transistors, it is naturally assumed that the gate voltage modifies the semiconducting SWNTs’ conductance. However, there is increasing evidence that Schottky barriers at the interface may play a central role [8–10], which affords new features, such as unusual transfer characteristics, temperature-dependent electrical transport, and the ability to apply effective dopants.

Scanning tunneling microscopy studies of SWNTs have reported peaks in the density of states (DOS) called Van Hove singularities (VHSs), which are believed to reveal the band structure and electrical properties of the SWNTs [11]. When the Fermi energy (*E*_F_) is close to the VHS, instabilities and divergences in the DOS occur, which can lead to the emergence of new phases of matter, such as superconductivity or magnetism [12, 13]. This suggests the possibility of engineering the material properties by modulating *E*_F_ and the VHS together. However, the position of the VHSs in the band structure of most materials cannot be changed. Instead, the carrier densities required to approach the VHSs can be reached by gating or chemical doping [14]. In previous studies, it was difficult to determine the region of gate voltage corresponding to VHSs in electrical transport measurements because the characteristic features of the conductance-versus-gate-voltage curve have not been identified.

In this work, we report a clear signature of VHSs in the conductance versus gate voltage of Schottky barrier transistors with individual suspended SWNTs. Critical saddle points were observed in regions of both positive and negative gate voltage, and the conductance flattened out when the gate voltage exceeded the critical value. These novel physical phenomena were confirmed when the temperature is below 100 K. Further, the temperature dependence of the electrical characteristics was also demonstrated in this type of Schottky barrier transistor.

## Experimental Section

The SWNTs were synthesized by floating catalytic chemical vapor deposition [15]. The ferrocene/sulfur powder, which used for catalyst source, was heated to 68 °C and flowed into the growth zone along with the mixed gas of 1000 sccm argon and 10 sccm methane. The deposited quantity of the isolated SWNTs can be controlled by the deposition time. Before fabricating the Schottky barrier transistors, a silicon wafer with a layer of silver was placed in the deposition zone for a specified time, and SWNTs were deposited on the surface of the silver film. By using this method, the quantity of isolated SWNTs deposited can be controlled by varying the deposition time. For the devices in this work, the deposition time was 5 s. Thus, individual SWNTs were isolated.

The Schottky barrier transistors were fabricated by a technique that we reported recently [16], which used the dynamic motion of silver liquid to suspend the SWNTs between prefabricated palladium electrodes during high-temperature annealing. First, a small quantity of SWNTs with a density of approximately a single SWNT per 5 µm was deposited onto the silver film, under which there were patterned palladium electrodes. When the temperature was raised above the melting point of the silver film (960 °C), the silver film became a silver liquid and moved toward the palladium electrodes. During this process, the SWNTs became suspended and strained between the palladium electrodes.

The electrode patterns were made using an electron beam lithography (Vistec EBPG 5000plus ES, Germany), and the devices were annealed in a chemical vapor deposition furnace. After that, the morphologies of the devices were characterized by scanning electron microscope (SEM, NOVA Nano SEM 430, USA). The low-temperature electrical transport properties of the devices were measured on a semiconductor characterization system (Keithley-4200), where the devices were placed in the liquid helium pot of a PPMS (PPMS-9, Physical Property Measurement System, Quantum Design Inc., USA) instrument.

## Results and Discussion

**Fig. 1:**
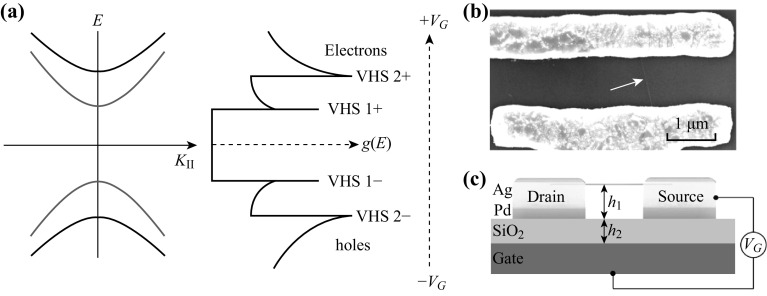
**a** Schematic of the electronic band structure (left) and DOS (right) of a semiconducting SWNT. Four sharp VHSs (VHS 1+, VHS 1−, VHS 2+, VHS 2−) appear at the onset of each subband. **b** Typical SEM image of a back-gate SWNT transistor. The SWNT is suspended, and the channel length is about 1 μm. **c** Schematic of the suspended SWNT transistor. The height of the suspended SWNT and the thickness of the insulating layer (SiO_2_) are 500 and 800 nm, respectively

The electrical properties of SWNTs are fundamentally dependent on their electronic band structure. The band structure is composed of multiple 1D subbands sliced from the Dirac dispersion cone of graphene [17]. Figure 1a shows the typical dispersion relations close to a Dirac point and the corresponding 1D DOS for semiconducting SWNTs. We argue that the Fermi energy of the electrons in the nanotube will be shifted by the gate voltage and will reach the energies corresponding to VHSs in the electronic DOS of the nanotube if the gate voltage is sufficiently large.

Figure 1b shows a SEM image of a back-gate SWNT transistor. An individual suspended SWNT is directly connected to two silver contacts and is capacitively coupled to the back gate, which can change the charge density on the FET. As shown in the schematic of the device in Fig. 1c, the SWNT is suspended and connected to the thin surface layer of the silver electrodes. The height of the suspended SWNT and the thickness of the insulating layer (SiO_2_) are 500 and 800 nm, respectively.

There are two main reasons for Schottky barrier formation at the silver/SWNT contact [8, 10, 18]. The first is a barrier created by the interface between the oxide layer of the silver and the SWNTs. Its resistance is a function of the overlap of the oxide layer and the SWNTs. This leads directly to a barrier between the SWNT and the electrode. The second reason is the difference between the work functions of silver and the SWNTs. The heights of the Schottky barrier for hole and electron injection depend on the band alignment at the interface. Our devices operate as unconventional Schottky barrier transistors, in which switching occurs primarily by modulation of the contact resistance rather than the SWNT conductance. These types of devices will exhibit new physical phenomena.

**Fig. 2:**
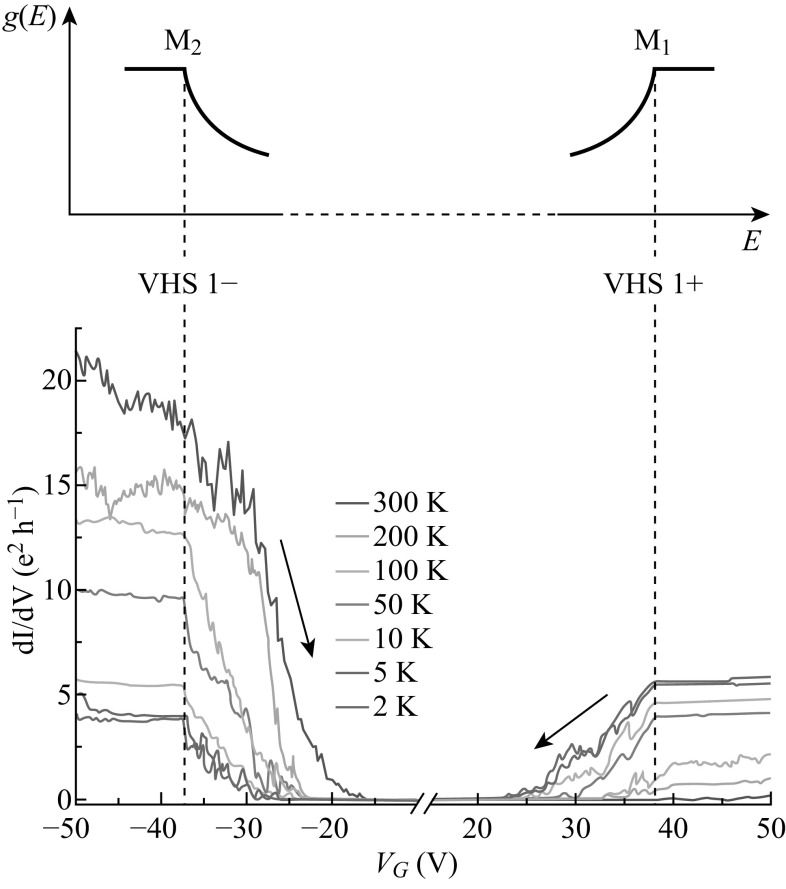
Differential conductance as a function of back-gate voltage for the suspended SWNT transistor at different temperatures. The black arrows indicate the sweep direction of the gate voltage. At temperature below 100 K, two critical saddle points appear at gate voltages of − 37.4 and 38.0 V, respectively. The two vertical dashed lines correspond to the onsets of the subbands

The differential conductance as a function of back-gate voltage for the suspended SWNT transistor at temperatures from 2 to 300 K is shown in Fig. 2. The source–drain bias is 100 mV. We did not measure zero-bias anomalies at the threshold, probably because the Schottky barriers at the Ag/SWNT contact yield low transparency. The gate voltages were swept from − 50 to 0 V and from 50 to 0 V, as indicated by the black arrows. At temperatures below 100 K, two critical saddle points appeared at gate voltages of − 37.4 and 38.0 V, respectively. When the gate voltage was swept from − 50 to 0 V, the conductance was flat with a constant small slope before the critical point (*V*_G_ = −37.4 V). After the critical point, the conductance decreased sharply and exhibited a rapid downward trend as the gate voltage approached zero. At low temperature (2 and 5 K), conductance oscillations are noticeable for gate voltages of − 37.4 V < *V*_*G*_ < 25 V and may be caused by Fabry–Perot interference [19]. When the temperature exceeded 100 K, the critical point and conductance plateau were not noticeable owing to thermal activation. In the positive gate voltage region, the critical point was at a gate voltage of 38.0 V, and the other characteristics were similar to those in the negative gate voltage region. Further, the device acted as an ambipolar FET at temperatures below 50 K and a *p*-type FET at temperatures above 100 K. The detailed physical mechanism is discussed below. The threshold voltages for the ambipolar FET are approximately − 21 and 23 V. The two vertical dashed lines in Fig. 2 correspond to the onsets of the subbands. We attribute the two critical points to VHS 1− and VHS 1+, respectively.

Previous studies demonstrated that the energy spacing of the VHSs in SWNTs depended mainly on their diameter [20]. For semiconducting SWNTs, the VHSs are separated from the Fermi energy by Δ*E* = *E*_G_/2 [15], where *E*_G_ is the energy gap. When the gate voltage is shifted from the threshold voltage by $e\Delta V_{\text{G}} = \Delta E/\alpha$, where *α* is the gate efficiency factor, the Fermi energy will be shifted to the VHSs. However, *α* cannot be directly measured and is related to the type and thickness of the dielectric layer.

Consider for simplicity a nondegenerate band *E*(*k*) and count the volume in *k*-space enclosed between the two surfaces. The corresponding DOS for this band is given by Ref. [21]:

1 $$g\left( E \right) = 2\int_{{E\left( \varvec{k} \right) = E}} {\frac{V}{{\left( {2\pi } \right)^{3} }}} \frac{{{\text{d}}S}}{{\left| {\nabla_{k} E\left( \varvec{k} \right)} \right|}}$$

where the factor *V*/(2π)^3^ gives the uniform density of allowed *k* vectors in *k*-space, *E*(*k*) = *E* is the constant-energy surface, and d*S* is the surface element of the equal-energy surface. Equation 1 clearly shows that singularities in the DOS are expected at the critical points, defined as those points in *k*-space for which

2 $$\nabla_{k} E(K_{0} ) = 0$$

At these points, the DOS is expected to exhibit anomalies as a function of energy. Near a critical point, we can expand the energy as a function of the wave vector. Indicating the principal axes of the quadratic form as *k*_*x*_, *k*_*y*_, *k*_*z*_ and taking the origin at the critical point itself, we have

3 $$E\left( k \right) = E_{c} \pm \frac{{\hbar^{2} }}{{2m_{x} }}k_{x}^{2} \pm \frac{{\hbar^{2} }}{{2m_{y} }}k_{y}^{2} \pm \frac{{\hbar^{2} }}{{2m_{z} }}k_{z}^{2}$$

where *m*_*x*_, *m*_*y*_, *m*_*z*_ > 0, and the plus or minus sign specifies the type of critical point. As shown in Fig. 2, the shapes of the VHSs better fit the saddle points *M*_1_ and *M*_*2*_, where *M*_1_ and *M*_2_ have one and two minus signs, respectively, and are thus two saddle points. We have

4 $$M1:\quad g\left( E \right) = C_{1} - V\frac{{\sqrt {2m_{x} m_{y} m_{z} } }}{{\pi^{2} \hbar^{3} }}\sqrt {E_{1} - E} \quad {\text{for}}\,\,E < E_{1}$$

5 $$M2:\quad g\left( E \right) = C_{2} - V\frac{{\sqrt {2m_{x} m_{y} m_{z} } }}{{\pi^{2} \hbar^{3} }}\sqrt {E - E_{2} } \quad {\text{for}}\,\,E > E_{2}$$

where *C*_1_ and *C*_2_ indicate constants or smoothly energy-dependent quantities.

Comparing Eqs. 4 and 5, we find that *g*(*E*) is still continuous at *E*_1_ or *E*_2_, but its slope changes discontinuously. The derivatives of the second terms with the square root of Eqs. 4 and 5 at *E*_1_ and *E*_2_ tend to positive infinity; as shown in Fig. 2, the critical saddle points *M*_1_ and *M*_2_ (corresponding to VHS 1+ and VHS 1−, respectively) appear in the conductance curves at low temperature. These types of VHSs are observed for the first time in the 1D carbon nanotube devices, mainly because of the peculiar properties of Schottky barrier transistors. Further, the suspended SWNTs acting as the channel in the transistor avoid the adverse effects resulting from the substrate [22–24], thus making it possibly to clearly identify the VHSs in the conductance-versus-gate-voltage curve. A previous work reported the signatures of VHSs in the conductance versus source–drain voltage and conductance versus gate voltage of carbon nanotube FETs [13]. When the voltage was decreased below the threshold value, the conductance increased to a certain value at *V*_G_ = −8 V and flattened out when the gate voltage was lowered further. They claimed that the steps in the conductance at *V*_G_ = −8 V might be due to the onset of the second subband in the nanotube. In our work, the critical points appeared at *V*_G_ ≈ ±38 V and were more obvious. The main reason for these differences is that in the previous work, the SWNTs lie on the substrate, and the distance between the SWNTs and the back gate is 400 nm [13], whereas in our devices, the SWNTs are suspended above the substrate, and the distance between the SWNTs and the back gate is approximately 1300 nm.

**Fig. 3:**
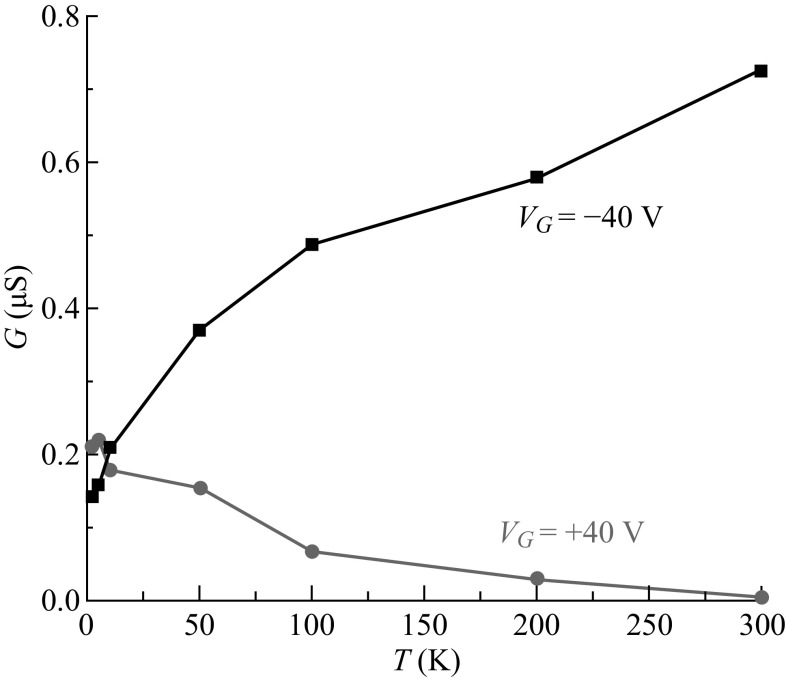
Drain conductance (*G*) at *V*_G_ = −40 V (black) and + 40 V (red) as a function of temperature

**Fig. 4:**
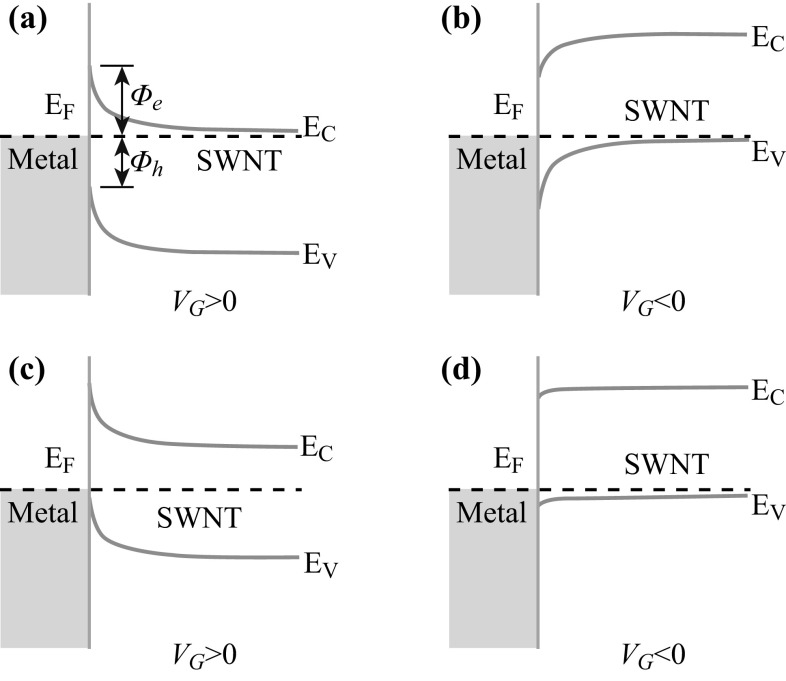
Schematics of the bands along the length of the SWNT at different temperatures and gate voltages. **a**, **b** At low temperature (*T* < 50 K), bands of the ambipolar SWNT device at *V*_G_ ≫ 0 (strong electron injection) and *V*_G_ ≪ 0 (strong hole injection). **c**, **d** At high temperature (*T* > 100 K), bands of the *p*-type SWNT device at *V*_G_ ≫ 0 and *V*_G_ ≪ 0. *Φ*_b_ shows the barrier height of the device

Figure 3 shows the drain conductance at *V*_*G*_ = −40 V (black) and + 40 V (red) as a function of temperature. In the hole injection region, the conductance increases with temperature, whereas the conductance decreases with increasing temperature in the electron injection region. Thus, the electrical characteristics of the transistors are converted from ambipolar to *p*-type with increasing temperature. Schottky barriers depend on the chemical nature of the metal, the adsorbed gases, and also the temperature. Further, not only the absolute value of the Schottky barrier heights but also their temperature coefficients are controlled by the detailed structure of the metal/semiconductor interface [25, 26]. There is no simple dependence between the height of Schottky barriers and their temperature coefficients. Schematics of the energy bands along the length of the SWNT are shown in Fig. 4. At temperatures below 50 K, the device is ambipolar, and the Schottky barrier allows injection of both electrons (Fig. 4a) and holes (Fig. 4b) at different gate voltages. The barrier heights for electron injection (*Φ*_e_) and hole injection (*Φ*_h_) are similar and are strongly modulated by the electrostatic field of the gate. As the temperature increases (> 100 K), the barrier height is larger for electron injection (*Φ*_e_) but smaller for hole injection (*Φ*_h_); thus, the transistor becomes *p*-type. As shown in Fig. 4c, d, when *V*_G_ > 0, it is difficult to achieve hole or electron injection, as the Fermi level is in the gap. When *V*_G_ < 0, hole injection is easily modulated by the gate voltage.

## Conclusions

A Schottky barrier transistor with a single suspended SWNT was fabricated using silver liquid dynamics. A clear signature of VHSs in the conductance versus gate voltage was observed in this Schottky barrier transistor. Critical saddle points appeared in regions of both positive and negative gate voltage, and the conductance flattened out when the gate voltage exceeded the critical value. These novel physical phenomena were evident when the temperature was lowered below 100 K. Further, the electrical characteristics of the transistors were converted from ambipolar to *p*-type with increasing temperature.
